# From Glycemic Control to Weight-Centered Therapy: A Paradigm Shift in Type 2 Diabetes and Its Public Health Promise

**DOI:** 10.3389/ijph.2026.1608891

**Published:** 2026-03-06

**Authors:** Yigitcan Sar

**Affiliations:** 1 Department of Pharmacology, Faculty of Pharmacy, Acibadem Mehmet Ali Aydinlar University, Istanbul, Türkiye; 2 Department of Pharmacology, Institute of Health Sciences, Istanbul University, Istanbul, Türkiye

**Keywords:** diabesity, diabetes mellitus, liraglutide, obesity, semaglutide

**The IJPH series “Young Researcher Editorial” is a training project of the Swiss School of Public Health.**

The future of type 2 diabetes mellitus (T2DM) management may not rest solely on controlling blood sugar but equally on addressing body weight and cardiorenal risk. T2DM has traditionally been managed through lifestyle interventions—such as dietary modification and regular physical activity—and pharmacological therapies including metformin, sulfonylureas, and insulin, all primarily aimed at achieving glycemic control. However, with the global obesity crisis—marked by the steadily rising prevalence of overweight (BMI 25–29.9 kg/m^2^) and obesity (BMI ≥30 kg/m^2^), now recognized as the second leading preventable cause of death—this glucose-centric strategy is rapidly evolving.

About 85% of individuals with T2DM are overweight or obese [1]. This statistic alone, derived from many epidemiological studies, establishes a strong association but does not prove a causal relationship. People with obesity are up to seven times more likely to develop T2DM than people without obesity; more than 40% of cases can be attributed to excess weight. Visceral fat accumulation triggers insulin resistance and β-cell dysfunction, the core pathophysiological processes underlying the disease. Obesity is thus not merely a comorbidity, but a fundamental factor associated with T2DM [2].

Paradoxically, weight gain can be exacerbated by some traditional glucose-lowering drugs including sulfonylureas and insulin, reinforcing a vicious cycle between obesity and poor metabolic control [3]. Against this growing global burden - affecting an estimated 589 million adults worldwide and projected to rise substantially by 2050 [4] - the limitations of weight - promoting therapies have highlighted the need for integrated metabolic strategies. In this context, the concept of “diabesity” has emerged, emphasizing the inseparable pathophysiological link between type 2 DM and obesity [3].

In response, novel pharmacotherapies, particularly glucagon-like peptide-1 receptor agonists (GLP-1 RAs) and sodium–glucose cotransporter-2 inhibitors (SGLT2i), have added weight loss to the therapeutic agenda, promoting it from a secondary observation to a recognized co-primary target alongside glycemic and cardiorenal risk reduction [5]. Large outcome trials show these agents not only achieve durable glycemic reductions and meaningful weight loss but also reduce major cardiovascular events and the progression of slow chronic kidney disease [1, 5]. Beyond these clinical outcomes, reorienting diabetes therapy toward weight-inclusive care has broader implications for public health.

Despite their clinical promise, GLP-1 RAs and SGLT2i have limitations. Adverse effects include gastrointestinal intolerance (nausea, vomiting), pancreatitis, and gallbladder disease, and their long-term safety profile is unknown. Sustaining real-world benefits over time is a challenge because the medications are expensive and tolerability issues often lead to treatment discontinuation [6].

Rapid uptake of these agents has created significant access and equity challenges. Liraglutide (Saxenda®) and semaglutide (Wegovy®) are FDA-approved for weight management in overweight and obese individuals without diabetes [7]. In parallel, semaglutide—originally approved for glycemic control in T2DM—has gained widespread off label popularity for cosmetic weight loss among non-diabetic populations [8]. In many low- and middle-income countries (LMICs), GLP-1 RAs can be purchased without a prescription, fueling unregulated use driven by aesthetic goals. Misuse, however, is not confined to LMICs: in high-income countries, telehealth platforms, online pharmacies, and lifestyle clinics have facilitated large-scale off-label prescribing, contributing to shortages observed in 2022–2023. These dynamics threaten prescribing integrity, complicate pharmacovigilance, and exacerbate global inequities [9, 10]. Social media has further amplified demand, with hashtags celebrating cosmetic weight loss garnering millions of views and glorifying rapid reductions while minimizing risks. This paradox—easy availability without prescription in some settings versus prohibitive costs in others—illustrates the fragmented global landscape of diabetes care.

While the scientific achievements behind GLP-1 RAs are extraordinary, their integration into real-world practice must be guided by evidence-informed policies, ethical oversight, and public education. International agencies such as the WHO could help harmonize access through initiatives like the Essential Medicines List, while national authorities must establish reimbursement frameworks, regulate marketing, and enforce prescription-only access. As early-career researchers in pharmacology and public health, we must advocate for regulation that prevents misuse, reimbursement models that prioritize need over profit, and health literacy strategies that counter misinformation. The recent lawsuits over GLP-1 RA adverse effects highlight the urgent need for transparent pharmacovigilance.

In sum, the pharmacological shift in T2DM management is evidence-based and promising, but its success will depend on responsible integration into practice and policy. Prioritizing weight as a co-primary therapeutic goal must go hand in hand with efforts to ensure equitable access, protect patients from exploitation, and safeguard public health. Innovation without access is inequity. Safety without supervision is illusion.

## Data Availability

The raw data supporting the conclusions of this article will be made available by the authors, without undue reservation.

## References

[1] Lazzaroni E Ben Nasr M Loretelli C Pastore I Plebani L Lunati ME Anti-Diabetic Drugs and Weight Loss in Patients with Type 2 Diabetes. Pharmacol Res (2021) 171:105782. 10.1016/j.phrs.2021.105782 34302978

[2] Chen X Zhang L Chen W . Global, Regional, and National Burdens of Type 1 and Type 2 Diabetes Mellitus in Adolescents from 1990 to 2021, with Forecasts to 2030: A Systematic Analysis of the Global Burden of Disease Study 2021. BMC Med (2025) 23:48. 10.1186/s12916-025-03890-w 39876009 PMC11776159

[3] Chawla R Jaggi S . Medical Management of Diabesity. J Assoc Physicians India (2019) 67(12):52–6. Available online at: https://pubmed.ncbi.nlm.nih.gov/31801332/ (Accessed May 27, 2025). 31801332

[4] International Diabetes Federation. IDF Diabetes Atlas. 11th ed. Brussels (BE): International Diabetes Federation (2025). Available online at: https://diabetesatlas.org/ (Accessed July 1, 2025).

[5] Mourougavelou V Chowdhury TA . Management of Hyperglycaemia in People with Obesity. Clin Med (Lond) (2023) 23(4):364–71. 10.7861/clinmed.2023-0135 37524409 PMC10541039

[6] Whitten R . Saxenda: A New Weight-Loss Drug for Obesity. Nurse Pract (2016) 41(5):18–21.

[7] Knudsen LB Lau J . The Discovery and Development of Liraglutide and Semaglutide. Front Endocrinol (Lausanne) (2019) 10:155. 10.3389/fendo.2019.00155 31031702 PMC6474072

[8] Bin Dayel FF Alanazi RJ Alenazi MA Alkhalifah S Alfaifi M Alghadeer S Evaluation of Pre-Treatment Assessment of Semaglutide Users: Balancing the Benefits of Weight Loss Vs. Potential Health Consequences. Healthcare (Basel) (2025) 13(15):1827. 10.3390/healthcare13151827 40805859 PMC12346479

[9] Shukar S Zahoor F Hayat K Saeed A Gillani AH Omer S Drug Shortage: Causes, Impact, and Mitigation Strategies. Front Pharmacol (2021) 12:693426. 10.3389/fphar.2021.693426 34305603 PMC8299364

[10] Thomsen RW Mailhac A Løhde JB Pottegård A . Real-World Evidence on the Utilization, Clinical and Comparative Effectiveness, and Adverse Effects of Newer GLP-1RA-Based Weight-Loss Therapies. Diabetes Obes Metab (2025) 27(Suppl. 2):66–88. 10.1111/dom.16364 40196933 PMC12000858

